# Adherence to Computer-Assisted Surgical Planning in 136 Maxillofacial Reconstructions

**DOI:** 10.3389/fonc.2021.713606

**Published:** 2021-07-16

**Authors:** Hongyang Ma, Sohaib Shujaat, Jeroen Van Dessel, Yi Sun, Michel Bila, Jan Vranckx, Constantinus Politis, Reinhilde Jacobs

**Affiliations:** ^1^ OMFS IMPATH Research Group, Department of Imaging & Pathology, Faculty of Medicine, KU Leuven, Leuven, Belgium; ^2^ Department of Oral and Maxillofacial Surgery, University Hospitals Leuven, Leuven, Belgium; ^3^ Department of Plastic, Reconstructive, and Aesthetic Surgery, University Hospitals Leuven, Leuven, Belgium; ^4^ Department of Dental Medicine, Karolinska Institutet, Stockholm, Sweden

**Keywords:** computer-assisted surgery (CAS), treatment adherence and compliance, patient-specific model, virtual surgical planning (VSP), 3D printing, oral and maxillofacial reconstruction, head and neck

## Abstract

**Objective:**

To investigate the adherence to initially planned maxillofacial reconstructions using computer-assisted surgery (CAS) and to identify the influential factors affecting its compliance for maxillofacial reconstruction.

**Patients and Methods:**

A retrospective analysis of 136 computer-assisted maxillofacial reconstructive surgeries was conducted from January 2014 to June 2020. The categorical parameters involved age, gender, disease etiology, disease site, defect size, bone flap segments, and flap type. Apart from descriptive data reporting, categorical data were related by applying the Fisher-exact test, and a p-value below 5% was considered statistically significant (P < 0.05).

**Results:**

The main reasons for partial or non-adherence included unfitness, patient health condition, and other subjective reasons. Out of the total patient population, 118 patients who underwent mandibular reconstruction showed higher CAS compliance (83.9%) compared to the 18 midface reconstruction (72.2%) without any statistically significant difference (p = 0.361). Based on the size of the defect, a significantly higher CAS compliance (p = 0.031) was observed with a minor defect (80.6%) compared to the large-sized ones (74.1%). The bone flaps with two or more segments were significantly (p = 0.003) prone to observe a partial (15.4%) or complete (12.8%) discard of the planned CAS compared to the bone flaps with less than two segments. The malignant tumors showed the lowest CAS compliance when compared to other disorders without any significant difference (p = 0.1).

**Conclusion:**

The maxillofacial reconstructive surgical procedures offered optimal compliance to the initially planned CAS. However, large-sized defects and multiple bone flap segments demonstrated a higher risk of partial or complete abandonment of the CAS.

## Introduction

Reconstructive maxillofacial surgery following tumor resection, trauma, osteonecrosis, and other infectious diseases is vital for restoring facial aesthetics, function, oral rehabilitation and improving the patient’s quality of life (QOL) (1). Depending on the complexity of the defect, the reconstruction might range from a local flap with secondary bone grafting to microvascular free flap surgery. The maxillofacial region mandates special care from a surgeon as it occupies a central position concerning the aesthetics and functionality, as an inadequate reconstruction might negatively influence the final outcome (2).

Previously, maxillofacial reconstruction with the traditional freehand technique offered a challenge for optimally repositioning the grafted segments and maintaining facial symmetry. However, with the advent of computer-assisted surgery (CAS) and three-dimensional (3D) printing, the reconstructive surgical accuracy and patient- and surgery-related outcomes have significantly improved (3, 4). Additionally, CAS has also played a vital role in improving the oral rehabilitation by increasing the predictability of replacing missing teeth with both first- and second-stage dental implant placement in the grafted region (5). Thereby, making CAS an indispensable tool for reconstructive surgery.

Over the past few years, the significant technological advancements and availability of surgeon-friendly software programs have led to the domination of CAS for maxillofacial reconstruction compared to its conventional counterpart by offering multiple advantages, which commonly include, improved resection accuracy, reduction in the operation, ischemia and hospitalization time, improved functional and aesthetic outcomes and minimization of the intersegmental gap size (6–8). At the same instance, the disadvantages such as preparation and planning time, and cost aspects cannot be ignored (9–11). Although, multiple centers now offer in-house CAS services for decreasing the time to therapy initiation (TTI) (12). However, an issue still exists where certain centers with low-volume of reconstruction cases rely on out-of-house services, which might cause a delay in the delivery and treatment time, in turn leading to further growth of the tumor (13). All these limiting CAS factors should be taken into consideration, as TTI has been known to be an influential factor for pathologic tumor upstaging, where an untimely intervention might lead to further tumor progression and increased mortality (14, 15).

Various studies have focussed on the accuracy and reproducibility of the CAS for maxillofacial reconstruction. However, a lack of evidence exists pertaining to the CAS compliance during the reconstructive procedures. It is questionable whether a surgeon completely adheres to the planned CAS (16). Previous studies reporting on the CAS compliance have only briefly reported whether the planning was executed entirely, partially, or abandoned and also failed to assess the factors which might influence its adherence.

Therefore, the present study was conducted to investigate the CAS compliance for initially planned maxillofacial reconstruction and to identify potential influential factors that might affect its adherence to the initially planned CAS.

## Material and Methods

**Figure 1:**
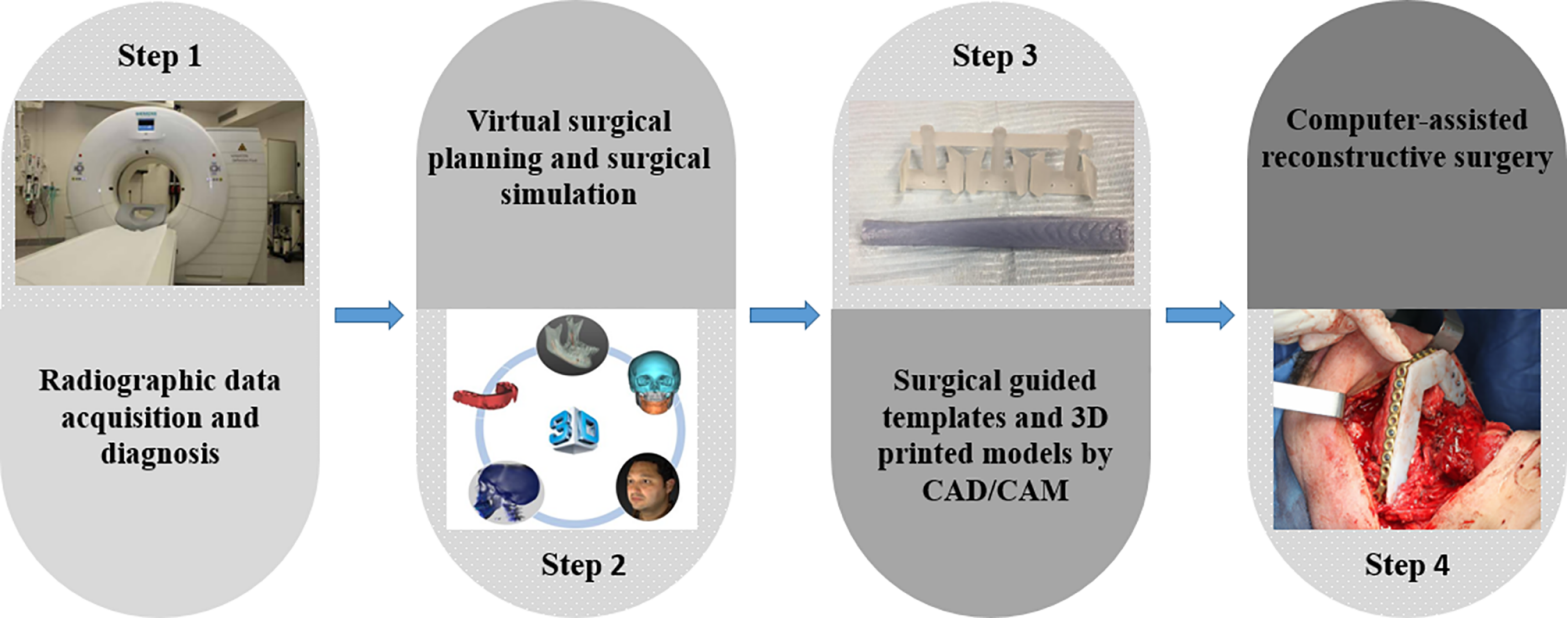
Workflow of Computer-assisted surgery in our single center.

The Local Ethics Committee approved the study (reference no.: S63615) and was conducted in compliance with the World Medical Association Declaration of Helsinki on medical research (clinicaltrials.gov, NCT04895319). A total of 210 patients who underwent CAS-based maxillofacial reconstruction were screened from January 2014 to June 2020. The inclusion criteria involved patients undergoing maxillofacial reconstruction with CAS, which included virtual surgical planning, CAD-CAM surgical guides/templates, and pre-bent plates on 3D printed models. The workflow in our single-center was illustrated in **Figure 1**. Reasons for reconstruction were oncologic, osteoradionecrosis, trauma, and osteoporosis. Patients undergoing computer-assisted implant surgery and orthognathic surgery were excluded.

**Figure 2:**
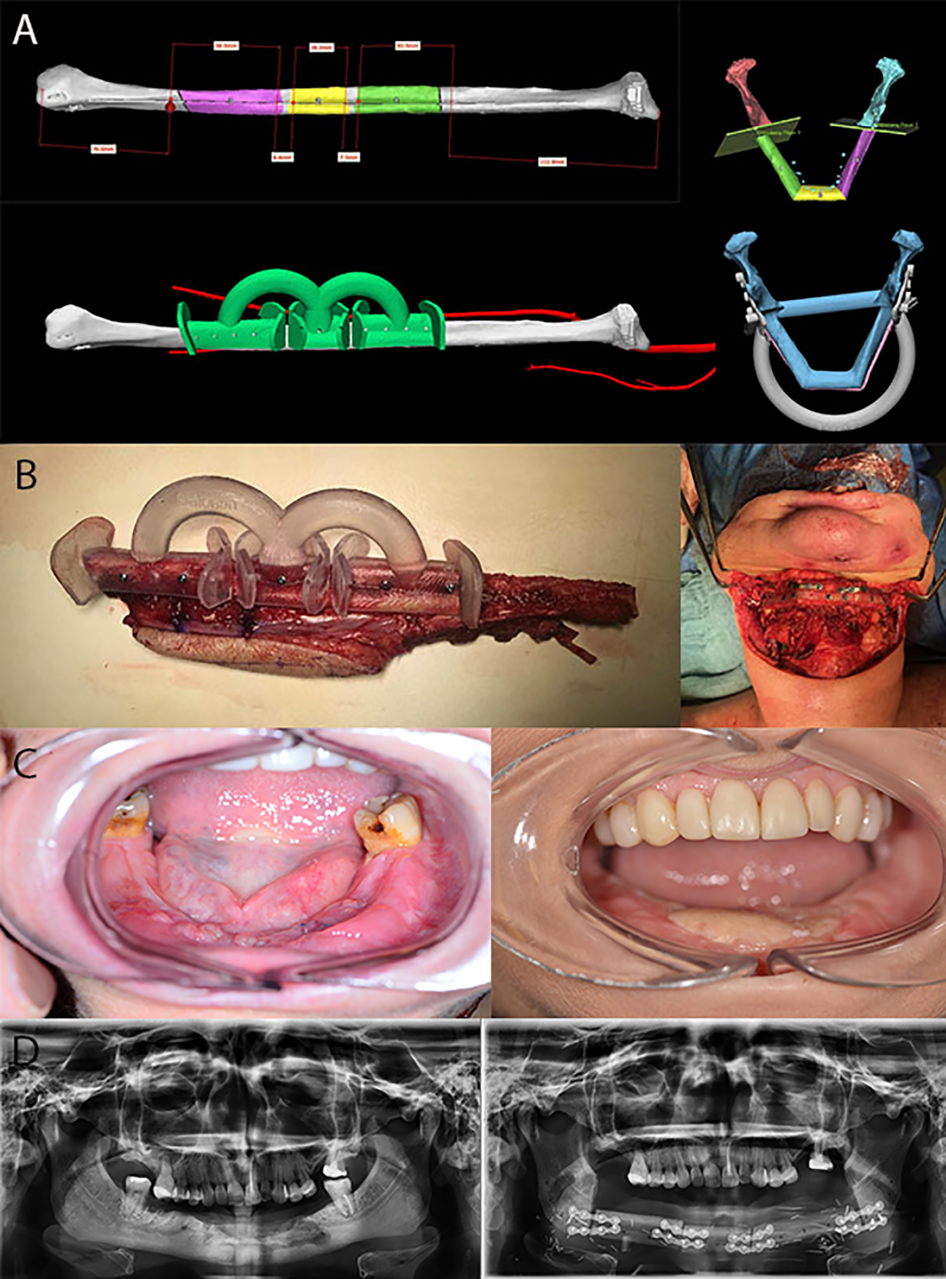
Computer-assisted surgical planning and execution for a squamous cell carcinoma reconstruction. **(A)** Preoperative virtual analysis and planning. **(B)** Fibular graft fabrication assisted by guided templates. **(C)** Preoperative and postoperative intraoral photos of squamous cell carcinoma resection with mandibular reconstruction. **(D)** Preoperative and postoperative panoramic radiographs.

All computer-assisted surgeries were planned by an experienced clinical engineer in discussion with the oral and maxillofacial surgical team. The virtual planning was performed to determine the resection, cut margins, and localize the optimal angles for performing osteotomies. After that, surgical cutting guides were designed utilizing a 3D designing software (3-Matic, Version 9.0-13.0, Materialise, Leuven, Belgium). The generated virtual templates and the planned 3D skeletal model were exported in a Standard Tessellation Language (STL) format and printed with a professional 3D printer (Connex 350 3D printer, Stratasys, Eden Prairie, MN, USA). The reconstructive plates were pre-bent on the 3D-printed model. A fixation tray was applied for the guided placement of the reconstructive plates. The screw holes’ locations were drilled and marked onto the surgical template by the surgeon (**Figure 2**).

**Figure 3:**
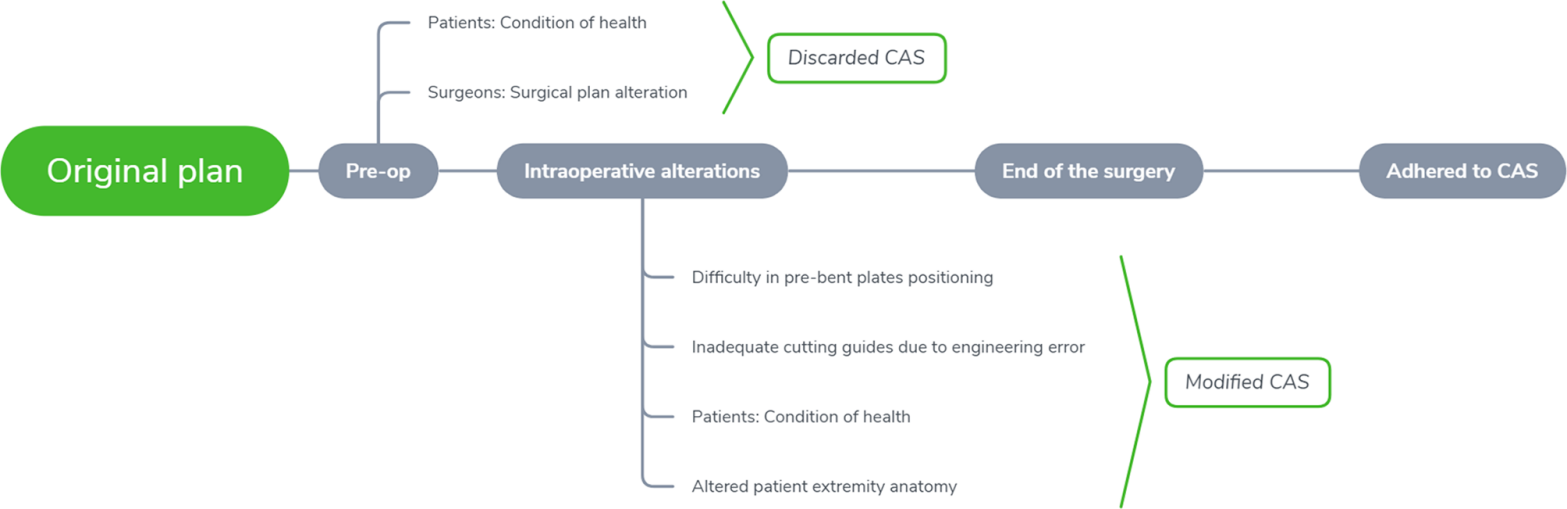
Flowchart of surgical adherence to computer-assisted surgery.

The patients were divided into three groups depending on the CAS compliance either during the pre-operative or intra-operatively, which included; complete adherence, partial adherence, and no adherence (**Figure 3**). The recorded categorical parameters involved disease etiology classified by either malignant or non-malignant tumor, disease site (mandible or midface), bone flap segments (< 2 or ≥ 2 segments), and flap type (bone flap or others). (The defects were classified based on Brown classification, where class I, II of mandibular defect and class I, II, V, VI of maxillary and midface defect were defined as a small defect; Class III, IV of the mandibular defect and class III, IV of maxillary and midface defect were defined as a large defect (17, 18).

### Statistical Analysis

Data were analyzed using IBM SPSS Statistics version 25.0 (IBM Corp., Armonk, NY: IBM Corp, USA). Mean values and standard deviation were recorded for all parameters. The categorical data were compared by applying the Fisher-exact test. A p-value below 5% was considered statistically significant (p < 0.05).

## Results

**Figure 4:**
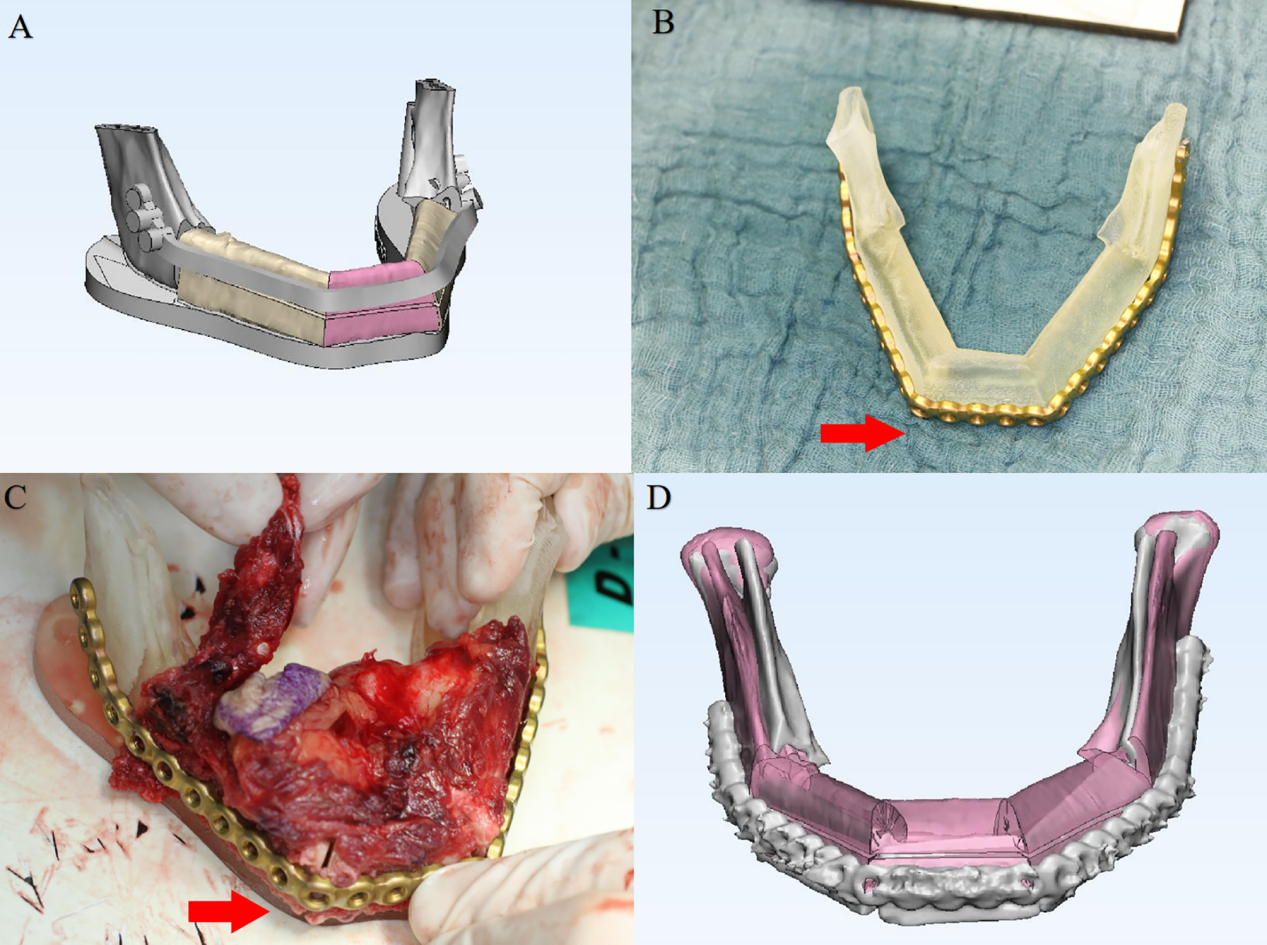
A 56-year-old patient with mandibular squamous cell carcinoma showing partial computer-assisted surgical compliance. **(A)** Virtual surgical planning for mandibular reconstruction. **(B)** Plate prebending on the 3D printed model. **(C)** Intra-operative plate bending modified due to unfitness. **(D)** Postoperative superimposition verifying the 3-D deviation of the reconstructed region compared with the original virtual surgical plan.

Following inclusion and exclusion criteria, clinical and image data of 136 consecutive patients (58 females, 78 males, mean age: 55.8 ± 18 years) undergoing CAS-based maxillofacial reconstruction were served further analysis. **Table 1** describes the patient- and surgery-related characteristics, where the majority of the patients were diagnosed with malignant tumor (n = 72) followed by maxillofacial trauma (n=16), benign tumor or odontogenic keratocyst (n=13), osteoradionecrosis (n=25) and temporomandibular joint ankyloses/congenital maxillofacial defect (n=10). The main reasons for partial abandonment of the planned CAS included unfitness of the cutting guide (n = 4) and pre-bent plates (n = 2), patients health condition (n=7). **Figure 4** illustrates an example of a case showing partial CAS compliance. In contrast, the complete discard of CAS was mainly attributed to subjective reasoning (**Table 2**).

**Table 1:** Patients characteristics.

Parameters	Classification	Numbers (N)	Percentage (%)
Gender (M/F)		78/58	57.4/42.6
Age (mean, SD)		55.8 ± 18	/
Adherence of CAS	Complete	112	82.4
	Partial	14	10.3
	Discarded	10	7.4
Etiology	Malignant tumor	72	52.9
	Benign tumor/cyst of jaw	13	9.6
	Trauma	16	11.8
	ORN	25	18.4
	Others	10	7.4
Disease site	Mandible	118	86.8
	Midface	18	13.2
Defect size	Small	72	52.9
	Large	64	47.1
Bone graft segments	0	20	14.7
	1	38	27.9
	2	39	28.7
	>2	39	28.7
Flap type	Fibula	88	64.7
	Iliac	22	16.2
	Scapula	6	4.4
	Plates or prosthesis only	20	14.7

CAS, Computer-assisted surgery; ORN, Osteoradionecrosis.

**Table 2:** Partially executed or discarded plan with reasons.

Influential factors	Reason	Numbers	Outcome
Unfitness	Guided templates	4	Partially executed plan
	Pre-bent plates	2	Partially executed plan
Patients’ health conditions	Tumor growth	2	Partially executed plan
	Tumor growth	1	Discarded plan
	Bone displacement	1	Partially executed plan
	Altered extremity	2	Partially executed plan
	Complex maxillary defect	1	Partially executed plan
	Death	1	Discarded plan
Subjective reasons	Surgical protocol changes	2	Partially executed plan
	Treatment plan alteration	3	Discarded plan
	Unaffordable cost	2	Discarded plan
	Patients’ non-compliance	3	Discarded plan

**Table 3** describes the factors influencing the compliance to the planned CAS. When evaluating the CAS compliance based on the defect site, patients who underwent mandibular reconstruction showed higher complete adherence (83.9%) compared to the midface reconstruction (72.2%) without any statistically significant difference (p = 0.361). Based on the size of the defect, a significantly higher conformity to the CAS (p = 0.031) was observed for patients with a minor defect (80.6%) compared to the large-sized ones (74.1%). The bone flaps with more than two segments were significantly (p=0.003) prone to observe partial (15.4%) or complete discard of the CAS (12.8%). The malignant tumors showed the lowest conformity to the CAS when compared to other disorders without any significant difference (p=0.1). As for the patients treated with a bone flap, complete adherence was significantly higher (85.3%, p=0.016) when compared with the non-bony flap group (65.0%).

**Table 3:** Influential parameters on the adherence of CAS.

Parameters	Classification	Total (n)	Complete adherence (n)	Percentage	Partial adherence (n)	Percentage	Not adherence (n)	Percentage	P-value
Site	Mandible	118	99	83.9%	11	9.3%	8	6.8%	0.361
	Midface	18	13	72.2%	3	16.7%	2	11.1%	
Defect size	Small	72	58	80.6%	6	8.3%	8	11.1%	0.031
	Large	64	99	74.1%	8	9.3%	2	6.8%	
Segments	<2	58	56	96.6%	2	3.4%	0	0.0%	0.003
	≥2	78	56	71.8%	12	15.4%	10	12.8%	
Aetiology	Malignant tumor	72	55	76.4%	11	15.3%	6	8.3%	0.1
	Non-malignant tumor	64	57	89.1%	3	4.7%	4	6.3%	
Flap type	Bone flap	116	99	85.3%	8	6.9%	9	7.8%	0.016
	Others	20	13	65.0%	6	30.0%	1	5.0%	

## Discussion

The present study explored the conformity to CAS for maxillofacial reconstructive procedures and investigated the influence of the parameters to identify the reasons it was partially executed or wholly discarded.

The present study’s findings suggested that the unfitness of the guided templates and patients’ health condition were most commonly observed in the partially abandoned CAS, whereas complete CAS discard was based on subjective reasoning. The factors which could have attributed to the reduced CAS compliance might include CT data segmentation accuracy, medical engineer proficiency, or precision of the printed stereolithographic model. Any error occurring due to the aforementioned factors would influence the CAS compliance. Besides, a prolonged waiting time for the surgery or an early CT scan in oncology patients caused the further growth of the malignant tumors, thereby requiring partial or complete discard of the plan. It should be kept that the CAS-based surgical planning and implementation only rely on the hard tissue, without considering the intra-operative influence of the soft tissue. The soft tissue and musculature have been known to forcefully position the bone flap in complex reconstructive procedures, which is not considered at the treatment planning phase and might lead to partial or complete discard of the CAS (19). Therefore, a surgeon should be aware of the biomechanical deformation of the soft tissue during CAS, and a patient-specific soft tissue predictive model should be generated based on the CT data, and finite element analysis at the planning phase improved planning (20).

Efanov et al. assessed the adherence to CAS for maxillofacial reconstruction and their findings were consistent with the results of the current study (21). However, their sample mostly involved orthognathic surgery patients, with only six patients requiring free tissue transfer, unlike our study where orthognathic surgical procedures were excluded to reduce the risk of bias. Hanken et al. reported a relationship between surgical accuracy and the number of bone flap segments for the maxillofacial reconstruction, where higher deviations occurred between virtual and real segment position in patients requiring reconstruction with two or three fibular or iliac crest segments compared to a single segment (22). The accuracy of CAS decreases with the increased number of segments, which might explain the partial adherence or complete discard. Previous evidence failed to report whether the defect size decreases the CAS compliance. Our findings suggested that a large-sized defect and increased bone segments were more prone to lower CAS compliance, especially in cases involving condylar region or mandibular angle where unfitness of pre-bent plates was mainly observed.

A variety of approaches can establish the improvement in CAS. Effective and constant communication between the surgeon and medical engineer might significantly improve the planned CAS. As the incomplete adherence not only leads to an increased risk of intra-operative complications but is also associated with higher financial costs if the plan is changed at the pre-operative stage (23). For improving the virtual planning and CAS, it is recommended to utilize a CT image with a slice thickness of less than 1mm and to advocate a professional 3D printing for printing the skull model to improve the contouring of the pre-bent plates (6). Another option could be the 3D printing of the patient-specific titanium plates which offers improved accuracy compared to the traditional pre-bent plates (24). Regarding the cutting guides, patient-specific titanium alloy cutting guides could be an alternative to improve fitness. These guides are thinner than the polyamide guides, allowing easier intraoral placement and decrease the amount of periosteal stripping and cutaneous resection (25).

The study had certain limitations. Firstly, the quantitative accuracy of the CAS was not assessed. Secondly, the retrospective nature of the study could have acted as a medium of bias. Thirdly, sample distribution was heterogeneous, mainly involving reconstruction following resection of the malignant tumors. Future studies should investigate the amount of error induced at each step of the planning to understand better and improve complex reconstructive procedures.

## Conclusion

CAS-based maxillofacial reconstructive surgery offered optimal conformity to the initially executed plan. However, large-sized defects and an increased number of bone flap segments led to a higher rate of partial or complete abandonment of CAS. Thereby, a surgeon should be aware of the possibility of non-adherence to the planned CAS for complex reconstructive procedures.

## Data Availability Statement

The original contributions presented in the study are included in the article/supplementary material. Further inquiries can be directed to the corresponding author.

## Ethics Statement

Written informed consent was obtained from the individual(s) for the publication of any potentially identifiable images or data included in this article.

## Author Contributions

HM and SS: study design, manuscript preparation, statistical analysis, data analysis, and interpretation. RJ and CP: study supervision. JD and YS: data collection. MB, JD, SS, JV, RJ, and CP: contributed to the manuscript review, critical revision for important intellectual content. All authors contributed to the article and approved the submitted version.

## Conflict of Interest

The authors declare that the research was conducted in the absence of any commercial or financial relationships that could be construed as a potential conflict of interest.
